# Atypical orbital mucosa-associated lymphoid tissue lymphoma involving the inferior rectus in a young adult: A case report and literature review

**DOI:** 10.1097/MD.0000000000047240

**Published:** 2026-01-23

**Authors:** Yin-Feng Wang, Hui-Chun Chen, Forn-Chia Lin, Chun-Hsiang Chang, Sheng-Min Hsu, Sheng-Chi Yang

**Affiliations:** a Education Center, National Cheng Kung University Hospital, College of Medicine, National Cheng Kung University, Tainan, Taiwan; b Department of Pathology, College of Medicine, National Cheng Kung University Hospital, College of Medicine, National Cheng Kung University, Tainan, Taiwan; c Division of Radiation Oncology, Department of Oncology, National Cheng Kung University Hospital, College of Medicine, National Cheng Kung University, Tainan, Taiwan; d Department of Ophthalmology, National Cheng Kung University Hospital, College of Medicine, National Cheng Kung University, Tainan, Taiwan; e Department of Ophthalmology, Tainan Hospital, Ministry of Health and Welfare, Tainan, Taiwan.

**Keywords:** case report, immunohistochemistry, lamina papyracea, magnetic resonance imaging, orbital lymphoma, radiotherapy

## Abstract

**Rationale:**

To report a rare case of orbital mucosa-associated lymphoid tissue (MALT) lymphoma presenting in a relatively young adult with inferior rectus involvement, and to review the literature on this atypical presentation.

**Patient concerns:**

A 46-year-old man presented with progressive swelling of the right lower eyelid, proptosis, and restricted eye movement.

**Diagnoses:**

Orbital magnetic resonance imaging revealed a well-defined, homogeneously enhancing mass involving the inferior rectus. Histopathology confirmed MALT lymphoma with immunophenotyping positive for CD20, BCL2, and myeloid cell nuclear differentiation antigen, and negative for CD5, CD10, and Cyclin D1.

**Interventions:**

The patient underwent orbital biopsy via the lamina papyracea, allowing safe access to the inferomedial orbit. He then received definitive radiotherapy (24 Gy in 16 fractions) following National Comprehensive Cancer Network guidelines.

**Outcomes:**

The patient showed a good early metabolic response to orbital radiotherapy on post-treatment positron emission tomography, without significant acute adverse effects, and was scheduled for ongoing surveillance.

**Lessons:**

This case illustrates an unusual demographic and anatomical presentation of orbital MALT lymphoma. The lamina papyracea route enabled high-quality tissue acquisition in a delicate periocular region, aiding accurate diagnosis. Literature review highlights the predominance of older female patients and superolateral orbital involvement, contrasting with this younger male case with inferior rectus infiltration.

## 1. Introduction

Lymphomas are a diverse group of hematologic malignancies originating from lymphoid tissue, with mucosa-associated lymphoid tissue (MALT) lymphoma representing a distinct, low-grade B-cell subtype commonly arising in extranodal sites.^[1]^ Although orbital involvement is uncommon among all lymphomas, orbital lymphoma is the most common primary orbital malignancy. MALT lymphoma is its predominant histologic subtype, accounting for 59% of orbital B-cell lymphomas.^[2]^

It typically presents as a slow-growing, painless mass in elderly individuals, often with a slight female predominance and preferential involvement of the superolateral orbit.^[2]^ Its rarity and nonspecific clinical and imaging features pose diagnostic challenges, as it mimics inflammatory, vascular, and benign neoplastic conditions.^[3]^ Early-stage MALT lymphoma is highly responsive to radiotherapy, which provides excellent local control.^[4]^ Accurate diagnosis relies on imaging, histopathology, and immunohistochemistry. Treatment typically follows standardized guidelines such as those from the National Comprehensive Cancer Network (NCCN).^[5]^ Despite its generally favorable prognosis, systemic evaluation remains essential to exclude disseminated disease, which directly influences staging and treatment strategies.^[2,5,6]^

Here, we presented a rare case of orbital MALT lymphoma in a 46-year-old male with inferior rectus involvement. And we aimed to describe the clinical presentation and diagnostic confirmation using an endoscopic endonasal biopsy, followed by definitive radiotherapy, and to review the relevant literature to contextualize this atypical presentation.

## 2. Case presentation

**Figure 1.**
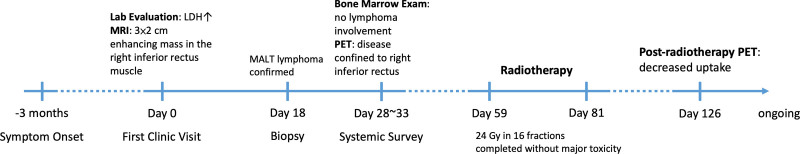
This timeline highlights the clinical presentation, diagnostic findings, and therapeutic management of the reported case. LDH = lactate dehydrogenase; MALT = mucosa-associated lymphoid tissue; MRI = magnetic resonance imaging; PET = positron emission tomography.

**Figure 2.**
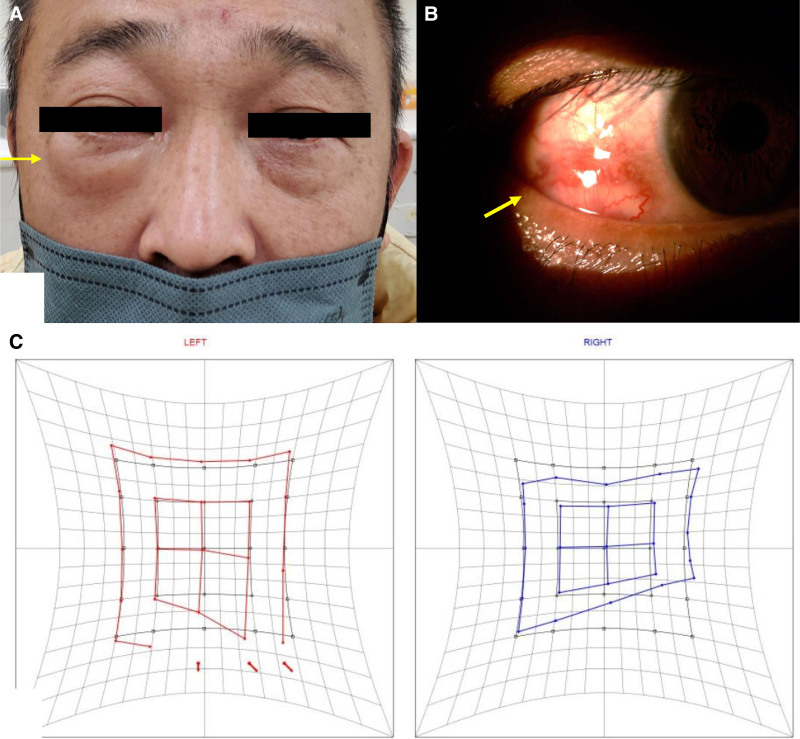
(A) Patient’s initial presentation, and arrow indicates right lower eyelid swelling. (B) Chemosis and engorged vessels of the conjunctiva (arrow). (C) Hess chart reveals right lower underaction in the right eye and right lower compensating overaction in the left eye.

A 46-year-old man without systemic diseases presented to the ophthalmology clinic with a 3-month history of progressive right lower eyelid swelling and proptosis (Figs. 1 and 2A). Upon examination, visual acuity was 20/25 in the right eye, with intraocular pressure elevated at 32 mm Hg. A palpable, ill-defined, non-tender mass was noted at the right lower eyelid, accompanied by temporal chemosis and engorged vessels in the conjunctiva (Fig. 2B). In addition, he reported limited extraocular movement, particularly in downward and inferolateral gaze, with slight upward limitation. Hess chart examination confirmed right inferior rectus underaction and contralateral synergist overaction (Fig. 2C).

**Figure 3.**
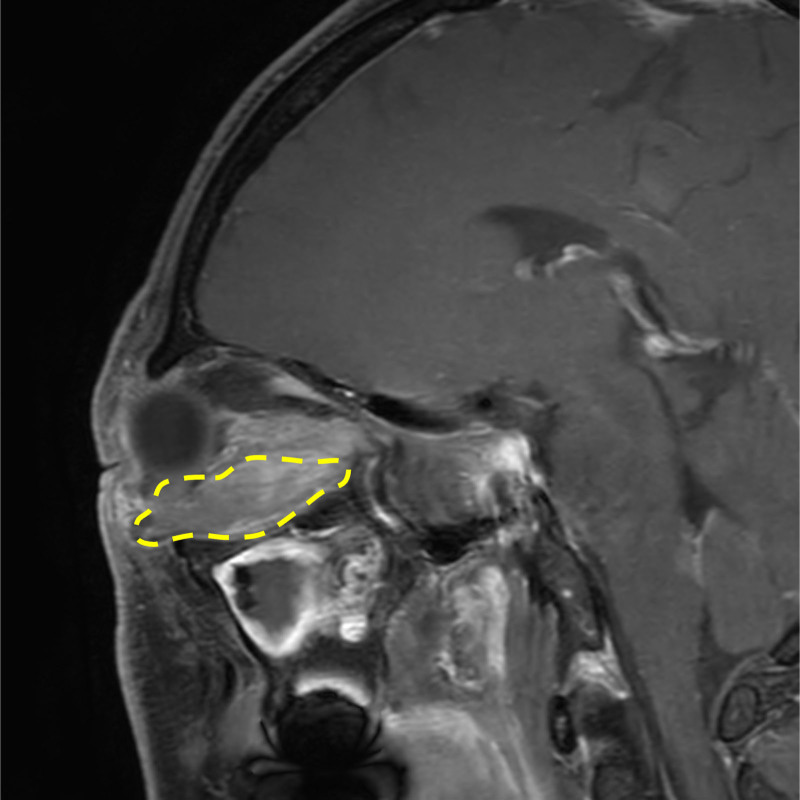
Sagittal T1-weighted image with contrast shows a 3 × 2 cm retrobulbar mass with homogeneous enhancement.

**Figure 4.**
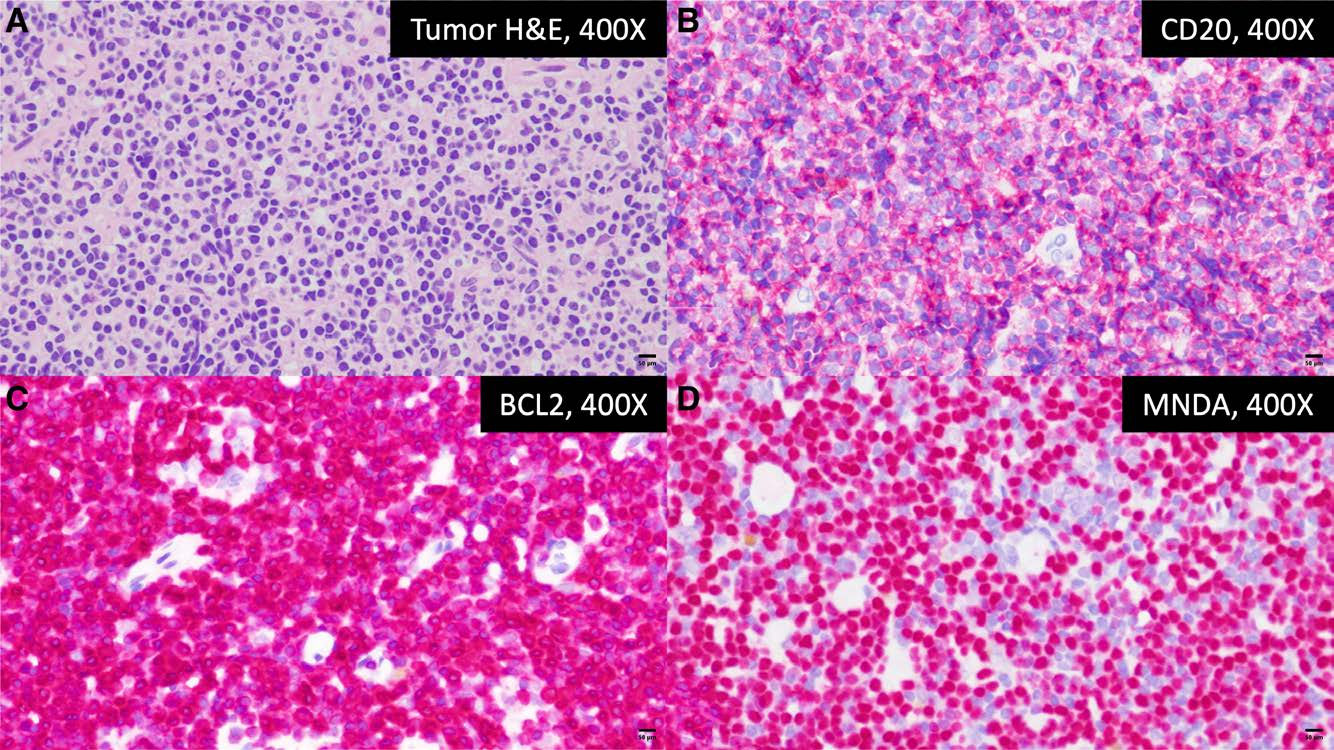
(A) Diffuse infiltrate of monocytoid lymphocytes with irregular nuclei and inconspicuous nucleoli (H&E, 400×). (B-D) The tumor cells are positive for CD20, BCL 2, and MNDA (immunohistochemistry, 400×).

Orbital magnetic resonance imaging (MRI) was performed to assess the lesion’s characteristics. T1-weighted MRI revealed a 3 cm × 2 cm isointense, homogeneously enhancing mass involving the right inferior rectus muscle (Fig. 3). Laboratory findings showed elevated lactate dehydrogenase. An orbital biopsy through the right lamina papyracea confirmed low-grade B-cell lymphoma with marginal zone phenotype, also known as MALT lymphoma. Histopathology revealed small tumor cells with irregular nuclear contours (Fig. 4A). Immunohistochemistry was positive for CD20, BCL2, and myeloid nuclear differentiation antigen (MNDA) (Fig. 4B–D), weakly positive for BCL6, and negative for CD3, CD5, CD10, CD23, Cyclin D1, IgD, and IRTA-1.

Staging evaluation with positron emission tomography (PET) scan showed metabolically active disease confined to the right inferior rectus muscle, without suspicious lymphadenopathy or distant extranodal involvement. Bone marrow biopsy revealed normocellular marrow without lymphoma involvement, confirming the absence of systemic spread. According to American Joint Committee on Cancer 8th Edition staging criteria,^[7]^ the patient was classified as stage, that is (T2N0M0, extranodal), indicating localized extranodal disease with tumor extension into the inferior rectus. There were no clinical or epidemiologic features raising suspicion for *Chlamydia psittaci* infection, and specific testing or empirical antibiotic therapy was therefore not pursued. He subsequently received radiotherapy to the right orbit with a total dose of 24 Gy in 16 fractions, in accordance with NCCN guidelines for localized orbital MALT lymphoma.^[5]^ Treatment was completed without significant adverse effects. An early post-treatment PET scan showed markedly decreased uptake in the right orbital muscles and no new abnormal hypermetabolic lesions, consistent with a good early metabolic response. Further clinical and imaging follow-up was arranged to monitor for relapse or systemic progression.

## 3. Discussion

Orbital tumors are rare, with an estimated incidence of 2.02 per million person-years.^[3]^ Among primary orbital malignancies, orbital lymphoma constitutes 55%, with MALT lymphoma represents comprising 59% of orbital B-cell lymphomas.^[2,6]^ Given this rarity and the wide differential of orbital lesions, accurate diagnosis relies on an integrated multimodal assessment combining clinical assessment, imaging, and tissue-based evaluation.^[3]^

Our case demonstrated classic clinical features of orbital MALT lymphoma, such as unilateral proptosis and gradual progression (mean symptom duration: 6.5 months).^[2]^ However, several atypical characteristics were observed. Most MALT lymphomas affect individuals over 50 years of age and show a slight female predominance (53%).^[2,6]^ In contrast, our patient was a 46-year-old male. Furthermore, although the superolateral orbit is most commonly affected,^[8]^ the lesion in this case involved the inferior rectus muscle, a distinctly uncommon site for orbital lymphoma. Extraocular muscle-centered lymphomas may mimic inflammatory or neurogenic disorders due to their deep anatomical location, often contributing to delayed diagnosis.^[9,10]^ Despite its unusual site, previously published series demonstrate high local control and minimal lymphoma-related mortality in orbital lymphomas with extraocular muscle involvement when treated with contemporary radiotherapy or chemoimmunotherapy.^[11]^ Similarly, in localized orbital MALT lymphomas treated with radiotherapy, only lacrimal gland involvement (rather than other orbital subsites) was independently associated with relapse.^[12]^ These findings collectively suggest that inferior rectus involvement alone does not confer a worse prognosis when the disease is localized and treated appropriately.

MRI findings of orbital lymphoma typically show isointensity to hypointensity on T1-weighted sequences and isointensity to hyperintensity on T2-weighted sequences, with homogeneous contrast enhancement: findings consistent with our case.^[8]^ Nonetheless, definitive diagnosis relies on histopathology and immunohistochemistry. Tumor cells demonstrated classic centrocyte-like morphology,^[13]^ and an immunophenotype positive for CD20, BCL2, and MNDA, and negative for CD3, CD5, CD10, CD23, and Cyclin D1, effectively excluding mantle cell and follicular lymphoma.^[2,5,14]^ CD5 negativity together with MNDA positivity are characteristic markers supporting MALT lymphoma and help distinguish it from more aggressive subtypes.^[15]^

Histologically, MALT lymphoma arises from the peri-follicular marginal zone and expands to efface surrounding tissue.^[13]^ While its morphology is consistent across anatomical sites, diagnostic accuracy can be limited in small or fragmented specimens. In this case, an endoscopic trans-lamina papyracea approach provided a direct and minimally invasive route to the inferomedial orbit, enabling retrieval of an intact specimen with preserved architecture: an important advantage in periocular lymphoid lesions.^[13]^ Compared with transconjunctival or external orbitotomy techniques, this approach minimizes external scarring and avoids manipulation of uninvolved orbital structures, although its utility is limited for superior or lateral lesions and requires proficiency in endoscopic techniques. Careful patient selection remains crucial, with ideal candidates presenting with inferomedial orbital involvement.^[16]^

The pathogenesis of MALT lymphoma is associated with chronic inflammation and antigenic stimulation.^[17]^ While *Helicobacter pylori* and *C psittaci* have been implicated in gastric and orbital MALT lymphomas respectively,^[18,19]^ the infectious etiology of orbital cases remains unclear. Autoimmune disorders such as Sjögren syndrome, together with elevated proinflammatory cytokines including interleukin-6 and tumor necrosis factor-α, promote B-cell activation and survival and may provide targets for immunomodulatory therapy.^[20,21]^ Known risk factors include older age, female sex, chronic local infection or inflammation, and underlying autoimmune diseases; however, many patients, particularly younger individuals, lack identifiable predisposing conditions, as seen in our case.^[2,19]^ Recent studies further highlight the contributions of cytokine networks and B-cell receptor-driven signaling to sustained lymphomagenesis. Aberrant nuclear factor kappa B activation, including pathways mediated by guanine nucleotide-binding protein-like 3-like protein, promotes pro-tumor B-cell proliferation.^[22]^ Furthermore, immunomodulators such as interleukin-27 receptor subunit alpha influence the tumor microenvironment and may serve as immune regulatory markers.^[23]^ Metabolic reprogramming, characterized by enhanced glycolysis and lipid synthesis, contributes to immune resistance and reflects a broader oncologic trend emphasizing inflammation-related mechanisms.^[24,25]^ In addition, dysregulation of the p53 pathway represents a complementary molecular vulnerability with emerging therapeutic relevance.^[26]^ Collectively, these contemporary insights broaden the immunopathogenic framework of orbital MALT lymphoma and underscore the interplay between inflammatory signaling, metabolic adaptation, and molecular survival pathways. Importantly, this immune-driven biology also provides a rationale for the established use of CD20-directed rituximab-based immunotherapy in recurrent disease, where it remains an effective systemic option.^[5,19]^

Accurate staging is essential for treatment planning. PET imaging and bone marrow biopsy confirmed localized disease in our patient, staged as, that is, under the American Joint Committee on Cancer system.^[7]^ Based on NCCN guidelines, he received definitive radiotherapy to the right orbit with a total dose of 24 Gy in 16 fractions, a regimen that remains standard for localized disease and achieves local control rates exceeding 90%.^[2,5]^ Although ultra-low-dose protocols (4 Gy in 2 fractions) have been explored for toxicity reduction, conventional dosing remains widely used with curative intent and was well tolerated in this case.^[5]^ Overall, orbital MALT lymphoma generally carries a favorable prognosis. Five-year survival for stage that is disease approaches 95%,^[2]^ although recurrence occurs in 10% to 15% of cases, highlighting the importance of long-term follow-up.^[5]^ In our patient, unilateral involvement, homogeneous contrast enhancement, and prompt treatment correspond with known favorable prognostic factors.^[4]^

## 4. Conclusion

This case underscores several clinical considerations. First, the demographic and anatomical deviations from typical presentations reinforce the need for diagnostic vigilance. Second, the lamina papyracea approach enabled safe and minimally invasive access to the inferomedial orbit, allowing for high-quality biopsy and accurate histologic diagnosis. Third, standardized RT remains effective in localized disease, and ongoing surveillance is essential due to potential of recurrence. Future advances may explore immunologic mechanisms to enhance therapeutic strategies beyond conventional radiotherapy.

## Author contributions

**Conceptualization:** Yin-Feng Wang, Sheng-Chi Yang.

**Data curation:** Yin-Feng Wang, Hui-Chun Chen, Chun-Hsiang Chang.

**Funding acquisition:** Sheng-Chi Yang.

**Investigation:** Yin-Feng Wang, Hui-Chun Chen, Forn-Chia Lin.

**Methodology:** Yin-Feng Wang, Sheng-Chi Yang.

**Project administration:** Yin-Feng Wang, Sheng-Chi Yang.

**Resources:** Forn-Chia Lin, Sheng-Min Hsu.

**Supervision:** Sheng-Min Hsu, Sheng-Chi Yang.

**Validation:** Yin-Feng Wang, Hui-Chun Chen, Forn-Chia Lin, Chun-Hsiang Chang.

**Visualization:** Yin-Feng Wang.

**Writing – original draft:** Yin-Feng Wang.

**Writing – review & editing:** Hui-Chun Chen, Forn-Chia Lin, Chun-Hsiang Chang, Sheng-Min Hsu, Sheng-Chi Yang.
